# The effects of Avemar treatment on feline immunodeficiency virus infected cell cultures

**DOI:** 10.1002/vms3.1141

**Published:** 2023-04-20

**Authors:** Katalin Réka Tarcsai, Máté Hidvégi, Oliga Corolciuc, Károly Nagy, Anna Anoir Abbas, Dharam V. Ablashi, Valéria Kövesdi, József Ongrádi

**Affiliations:** ^1^ Doctoral School Semmelweis University Budapest Hungary; ^2^ Jewish Theological Seminary – University of Jewish Studies (OR‐ZSE) Budapest Hungary; ^3^ Department of Transfusion Medicine Semmelweis University Budapest Hungary; ^4^ Molecular Microbiology Diagnostic Laboratory Eötvös Lóránd University (ELTE) Budapest Hungary; ^5^ HHV‐6 Foundation Santa Barbara California USA; ^6^ Department of Public Health Semmelweis University Budapest Hungary

**Keywords:** AIDS, Avemar pulvis, destruction of retrovirus carrier cells, feline adenovirus, feline immunodeficiency virus, fermented wheat germ extract, inhibition of virus release

## Abstract

**Introduction:**

In addition to standard highly active antiretroviral therapy protocols, complementary therapies using natural compounds are widely used by human immunodeficiency virus (HIV)‐infected human patients. One such compound is the fermented wheat germ extract (FWGE), named Avemar.

**Materials and methods:**

In this study, we investigate the effects of Avemar in a feline‐acquired immunodeficiency syndrome model. MBM lymphoid cells were acutely infected by the American feline immunodeficiency virus (FIV)‐Petaluma (FIV‐Pet) and the European FIV Pisa‐M2 strains. FL‐4 lymphoid cells, continuously producing FIV‐Pet, served as a model for chronic infection. Crandell Rees feline kidney (CRFK) cells were infected by either FIV‐Pet or feline adenovirus (FeAdV) as a model for transactivation and opportunistic viral infection. Cell cultures were treated pre‐ and post‐infection with serial dilutions of spray‐dried FWGE (Avemar pulvis, AP), a standardized active ingredient in commercial Avemar products. Residual FIV and FeAdV infectivity was quantified.

**Results:**

In a concentration‐dependent manner, AP inhibited replication of FIV strains in MBM and CRFK cells by 3–5 log. Low AP concentration prevented FIV‐Pet release from FL‐4 cells. Higher concentrations destroyed virus‐producing cells with cytopathic effects resembling apoptosis. AP strongly inhibited FeAdV production inside CRFK cells but not in HeLa cells. Adenovirus particles are then released via the disintegration of CRFK cells.

**Discussion:**

This report is the first to describe the antiviral effects of Avemar. Further studies are required to confirm its in vitro and in vivo effects and to investigate the potential for its use as a nutraceutical in FIV‐infected felines or HIV‐infected humans.

**Conclusion:**

Avemar, as a single nutraceutical, inhibits FIV replication and destroys retrovirus carrier cells. An important conclusion is that prolonged Avemar treatment might reduce the number of retrovirus‐producing cells in the host.

## Graphical abstract

The antiviral effect of a fermented wheat germ extract, named Avemar, was tested in vitro in the feline AIDS model. In a dose‐dependent manner, Avemar inhibited the replication of both European and American FIV strains in feline lymphoid cell cultures. Avemar destroyed chronically FIV‐producing lymphoid cells with cytopathic effects resembling apoptosis. Avemar inhibited feline adenovirus replication in CRFK cells but did not in cancerous HeLa cultures. The possible molecular mechanisms of antiviral effects are discussed. Further studies are recommended to investigate its potential for use as a nutriceutical in FIV‐infected felines and HIV‐infected humans.

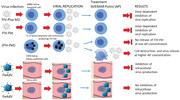

## 1 INTRODUCTION

The immune system's response to human immunodeficiency virus (HIV) infection leads to metabolic changes and weight loss with significant morbidity and mortality. Highly active antiretroviral therapy (HAART) has significantly modified the natural course of acquired immunodeficiency syndrome (AIDS), transforming it into a chronic disease; however, cells carrying integrated HIV cannot be eradicated by HAART, and a small fraction of the virus escapes from control. AIDS‐related diseases are compounded by ageing‐associated comorbidities, including non‐AIDS‐defining malignancies (Dalzini et al, 2020). So far, the search for drugs capable of selectively eradicating HIV from humans or feline immunodeficiency virus (FIV) from cats has failed.

HIV‐infected patients frequently use complementary and alternative medicine therapies to improve nutritional status, treatment outcomes and alleviate the side effects of antiretroviral therapy. A proprietary fermented wheat germ extract (FWGE), known as Avemar, is a widely used immunomodulatory and anticancer nutraceutical (Boros et al., 2005). Avemar has been also used in the veterinary practice under different formulations, Immunovet for farm animals and pets or, for horses, eCalm (Stipkovits et al., 2004, Sutton et al., 2018). It is produced under good manufacturing practice conditions (Hidvégi, Rásó, Tömösközi‐Farkas, Szende, et al., 1999). Avemar pulvis (AP, FWGE powder), the active ingredient, is standardized to methoxy‐substituted benzoquinone marker compounds, that is 2,6‐dimethoxy‐*p*‐benzoquinone (2,6‐DMBQ) and 2‐methoxybenzoquinone (2‐MBQ). These compounds are liberated as aglycones by the action of glycosidases of the fermenting organism, *Saccharomyces cerevisiae*. AP contains all the water‐soluble materials of fermented wheat germ liquid, encapsulated with maltodextrin and spray‐dried. A single dose (17 g) of the commercial Avemar granulate contains 8.5 g AP plus added flavouring ingredients. FWGE is a complex mixture of thousands of molecules. Most of them have not been chemically identified yet (Telekes et al., 2009). Avemar has a direct pro‐apoptotic effect in lymphoma cells and boosts host immune effector mechanisms (Barisone et al., 2018). It initiates intrinsic mitochondrial‐dependent apoptotic signalling in tumours (Bencze et al., 2020), restores normal immune functions in immunocompromised animals (Hidvégi, Rásó, Tömösközi‐Farkas, Lapis, et al., 1999), reverses the transformed metabolic phenotype (Boros et al., 2005), inhibits ribonucleotide reductase (Saiko et al., 2007), cleavages poly(ADP)ribose polymerase (PARP) (Comin‐Anduix et al., 2002) and shows robust antiproliferative effects and triggers tumour cell death through apoptosis (Zhurakivska et al., 2018). AP has a firmly established safety profile (Heimbach et al., 2007) and an independent panel of medical, food safety and toxicology experts confirmed Avemar's generally recognized as safe status in accordance with US FDA's regulations (Hoffman et al., 2005). In countries of the European Union, Avemar has been approved as a dietary food for cancer patients and has since been marketed as a non‐prescription medical nutriment in other parts of the world (Patel, 2014). In cancer and autoimmune clinical studies, Avemar has proven to be advantageous in terms of disease progression, overall survival and clinical outcome (Mueller & Voigt, 2011).

FIV‐infected cats can serve as informative animal models for AIDS studies (Matteucci et al., 1995). FIV is a widespread pathogen of domestic cats (Pedersen, 1993). Feline isolates are classified into subtypes A (USA‐California and Europe), B (Europe, Japan, and USA except for California), C (Canada) and D (Japan) (Hohdatsu et al., 1996). So far, FIV infection has been detected in 37 felid species living free or in zoological collections (O'Brien et al., 2012). FIV‐infected cats are susceptible to opportunistic infections and cancer development, thus offering a strong parallel to related virus‐based pathologies in humans. Consequently, viral interactions, both related to AIDS and to age‐related complications, can be studied in the feline AIDS model (Ongrádi et al., 2013).

Adenoviruses (AdVs) are widespread among mammals and can cause severe infections. In human AIDS, early genes (E1A, E1B) of AdVs have been shown to transactivate HIV and consequently promote AIDS progression (Kliewer et al., 1989). AdVs, reactivated from latency, elicit opportunistic infections. Before the introduction of HAART, approximately 20% of AIDS patients died of untreatable gastroenteritis. Species C AdV types are the more frequent causative agents, but unusual B and D intratypic recombinants are also produced and excreted in the stool and urine of AIDS patients (Hierholzer, 1992). After bone marrow or hematopoietic stem cell transplantation, reactivated AdVs can elicit prompt lethal complications or lifelong sequelae (Lion, 2019). Inhibition of AdV expression might provide clinical benefits to AIDS patients. AdV infections have not been widely studied in cats and Felidae. We first described European and partially American AdV epidemiology and characterized the first feline adenovirus (FeAdV) isolate. It is related to but distinct from human adenovirus 1 (Ongrádi et al., 2019).

In the present paper, our studies on Avemar in the feline AIDS model are presented.

## 2 MATERIALS AND METHODS

### 2.1 Avemar pulvis

AP and Avemar granulate were obtained from the manufacturer (Biropharma Kft., Kunfehértó, Hungary). The samples were freshly dissolved in sterile phosphate‐buffered saline at 10 mg/mL concentration, cleared by centrifugation, filtered through a 22 μm filter and stored in the freezer at −20°C. Further dilutions were prepared in complete Dulbecco's modified Eagle's medium (DMEM) or in complete RPMI‐1640 medium, depending on the culture conditions of the particular cell line studied.

### 2.2 Cells and viruses

MBM feline lymphoid cells were maintained in suspension as previously described (Matteucci et al., 1995). FL‐4 feline lymphoid cells chronically producing the Californian isolate FIV‐Petaluma (FIV‐Pet) without cytopathic effect (CPE) (FL‐4‐Pet) were maintained as previously described (Yamamoto et al., 1991). Crandell Rees feline kidney (CRFK) epithelial cells (ATCC CCL‐94) and HeLa human cervical carcinoma cells (ATCC CCL‐2) were maintained as previously described (Ongrádi et al., 2019) (Figure S1). Besides the FIV‐Pet (Clade A) (Hohdatsu et al., 1996) containing supernatant of FL‐4 cells, a European isolate FIV Pisa‐M2 (FIV‐M2, Clade B) was used. The FeAdV (ATCC VR‐1890) (Ongrádi et al, 2019) was used throughout this study (Figure S2).

AP stock media was confirmed to be free from bacterial lipopolysaccharide (Pyrogen plus single test kit, Cambrex Bio Science, Walkersville, MD, USA). Cells were confirmed to be free of intracellular feline leukaemia virus p27, using the methodology previously described (Matteucci et al., 1995) and mycoplasma contamination (MycoProbe Mycoplasma detection kit, R&D Systems, Minneapolis, MN, USA, or using mycoplasma detecting DAPI, Roche Diagnostics GmbH, Mannheim, Germany) (Ligasová et al., 2019).

### 2.3 FIV stocks and virus titration

A stock solution of FIV‐M2 was obtained by infecting MBM cells with FIV‐M2. The supernatant fluid of FL‐4 cells was used as a source of FIV‐Pet. On day 10, viruses were harvested in the supernatant fluid by centrifugation and stored as 1 mL aliquots at −80°C. Samples were titrated on CRFK cells as described (Yamamoto et al, 1991, Matteucci et al, 1995). Median tissue culture infective dose (TCID_50_) values were determined by the Reed–Muench method (Reed & Muench, 1938), FIV‐Pet titred at 10^5^ syncytium forming unit/mL (SFU/mL), whereas FIV‐M2 did so at 10^4^ SFU/mL. FIV production was quantitated by p24 antigen ELISA (Quick Titer FIV Lentivirus Quantitation Kit, FIV p24 ELISA, Cell Biolabs, Inc., San Diego, CA, USA). FeAdV stocks were produced in HeLa cultures. Clarified supernatants of cells were purified and concentrated (ViraBind Adenovirus Purification Kit, Cell Biolabs, Inc., San Diego, CA, USA) according to the manufacturer's instructions. Virus stocks were stored at −80°C. At 48 h post‐infection (pi), intracellular hexon antigens were detected by immunocytochemistry (ICH, Quick Titer Adenovirus Titer Immunoassay Kit, Cell Biolabs, Inc., San Diego, CA, USA) as previously described (Ongrádi et al., 2019). The final concentration of the virus was 1.35 × 10^7^ infectious unit/mL (IU/mL).

### 2.4 Monitoring the effect of Avemar pulvis on cell cultures

Cell growth, CPE and viability determined by trypan blue exclusion were monitored daily by light microscopy. Cytotoxicity of AP, as determined by the growth arrest of the cells in the logarithmic phase, was determined by EZ4U, a fourth‐generation, non‐radioactive cell proliferation and cytotoxicity assay (Biomedica Medizinprodukte GmbH, Wien, Austria). MBM, FL‐4, CRFK and HeLa cells were aliquoted with serial AP dilutions at final concentrations between 0 and 5000 μg/mL. Samples containing 1 × 10^4^ cells were collected on days 1, 2, 3 and 7 and complemented with 20 μL substrate (tetrazolium). Optical density was read as above. The ratio of living cells was calculated relative to untreated controls (%).

### 2.5 Simultaneous FIV infection and Avemar pulvis treatment of feline cells

MBM cells at 4 × 10^4^ quantity per well in 100 μL medium were seeded in 96‐well plates in triplicate and subsequently infected by 10^5^ TCID_50_ FIV‐Pet or FIV‐M2 in 50 μL medium. Infected cells were treated with serial dilutions (final concentrations between 0 and 1000 μg/mL) of 50 μL AP at −2, −1, 0, +1 and +2 h relative to FIV infection, in triplicate. Cell morphology and viability were serially monitored. At various time intervals, *t* = 0, 5, 7, 8 and 11 days, 20 μL cell suspensions at a concentration of 1 × 10^6^/mL were removed from the wells and spotted onto Teflon‐coated multiwell slides. After air‐drying, methanol fixation and Giemsa staining, photographs were taken at a magnification of ×200 (data not shown). Cell‐free supernatants were obtained at time intervals, *t* = 0, 5, 7, 8 and 11 days, and titrated on CRFK cells. In parallel experiments, 4 × 10^4^ CRFK cells in 100 μL complete DMEM were aliquoted in 96‐well plates in triplicate. The next day, their medium was replaced with 100 μL FIV‐Pet inoculum at 10^5^ TCID_50_/mL. After incubation at 37°C for 4 h, their medium was supplemented with 100 μL AP dilutions (final concentrations between 0 and 1000 μg/mL). CPE was monitored daily, and cell‐free supernatants were removed on days 0, 5 and 9 to quantitate their p24 content by ELISA. In a separate experiment, 4 × 10^4^ FL‐4 cells in 100 μL complete RPMI‐1640 medium were seeded in 96‐well plates. At 4‐h pi, 100 μL AP dilutions (final concentrations between 0 and 1000 μg/mL) were added to the wells in triplicate. On day 8, supernatants and cells were separated by centrifugation; pellets were frozen and thawed three times and resuspended in 100 μL complete RPMI‐16410 medium. FIV‐Pet content in supernatants and pellets was titrated on CRFK cells. Morphology and viability of FL‐4 cells were monitored as described for MBM cells above. After air‐drying, methanol fixation and Giemsa staining, photographs were taken at a magnification of ×200, at time points detailed in Figure 3. Further magnification was obtained by using the optical and digital zoom functions of the camera.

### 2.6 FeAdV infection and Avemar pulvis treatment of HeLa and CRFK cells

HeLa and CRFK cells were aliquoted at 25 × 10^4^/mL in 1 mL medium in 24‐well plates. After 1 h, the cells were treated in triplicate with 500 μL AP solutions at final concentrations of 0, 10, 50, 100, 250, 500 or 1000 μg/mL, respectively. After 1 or 24 h, the cells were infected with 2.5 × 10^4^, 25 × 10^4^, 50 × 10^4^ or 25 × 10^5^ IU/mL (moi 0.1, 1, 2, and 10) FeAdV in 100 μL, in triplicate. In simultaneous experiments, cells first were infected with FeAdV, as above, and subsequently treated with AP solution in dilutions as above. Controls contained either AP dilutions or FeAdV only. At 48 h pi, single cells exhibiting FeAdV infection were visualized in five randomly selected microscopic fields by immunoassay. The ratio of infected and treated cells was expressed in percentage relative to virus control. Supernatant samples were simultaneously removed from infected cells, and their residual FeAdV infectivity was titrated on HeLa cells as previously described (Ongrádi et al., 2019).

### 2.7 Statistical analysis

All experiments were carried out in triplicate or quadruplicate. The data were expressed as the mean ± standard error of mean. Statistically significant differences were evaluated by a two‐sample *t* test (*p* < 0.05 was deemed to be significant).

## 3 RESULTS

### 3.1 Cell growth arrest and cytotoxicity elicited by Avemar pulvis

**FIGURE 1:**
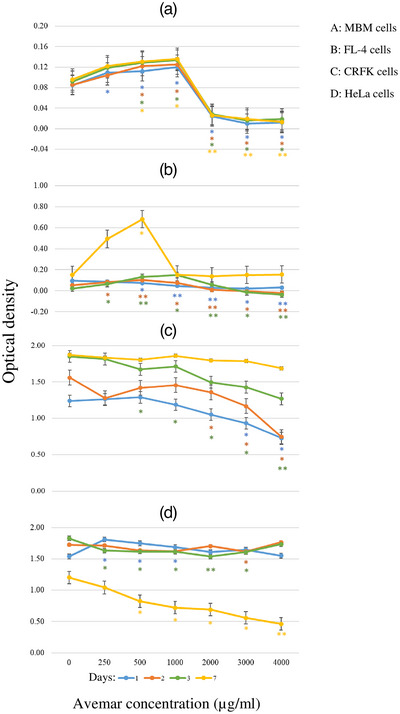
Effect of a single dose of Avemar on the replication of MBM, (a) FL‐4, (b) Crandell Rees feline kidney (CRFK), (c) HeLa and (d) cells. Aliquots of cells were removed on days 1, 2, 3 and 7, and the ratio of living cells was determined by optical density. *p < 0.05, **p < 0.001.

The effects of serial dilutions of AP on the replication, viability and morphology of cells were monitored daily. AP of 5000 μg/mL exerted strong cytotoxicity on all cell types (data not shown). MBM cells were stimulated by low AP concentrations (up to 1000 μg/mL) up to day 7, but from this concentration onwards, a sharp onset of cytotoxicity was demonstrated (Figure 1A). A smaller fraction (13%–17%) of cells did not grow but remained viable as demonstrated by trypan blue (data not shown). During the first days of culture, the replication of FL‐4‐Pet cells (Figure 1B) was slightly increased by AP up to 1000 μg/mL. Low concentrations of AP had minimal effect on CRFK cells (Figure 1C), which formed a monolayer by day 3, similar to negative control samples. AP, administered at concentrations higher than 2000 μg/mL, diminished CRFK cell growth with surviving cells regaining their replicative capacity. The subconfluent HeLa culture (Figure 1D) remained discontinuous, and cell growth stopped until day 3. Furthermore, a marked dose‐dependent growth arrest on HeLa cells was seen by day 7, suggesting that the onset of AP cytotoxicity started between 1000 and 2000 μg/mL concentration. The antiviral effect of AP was thus tested to a maximum concentration of up to 1000 μg/mL.

### 3.2 Effect of Avemar pulvis on FIV‐infected MBM cells

**FIGURE 2:**
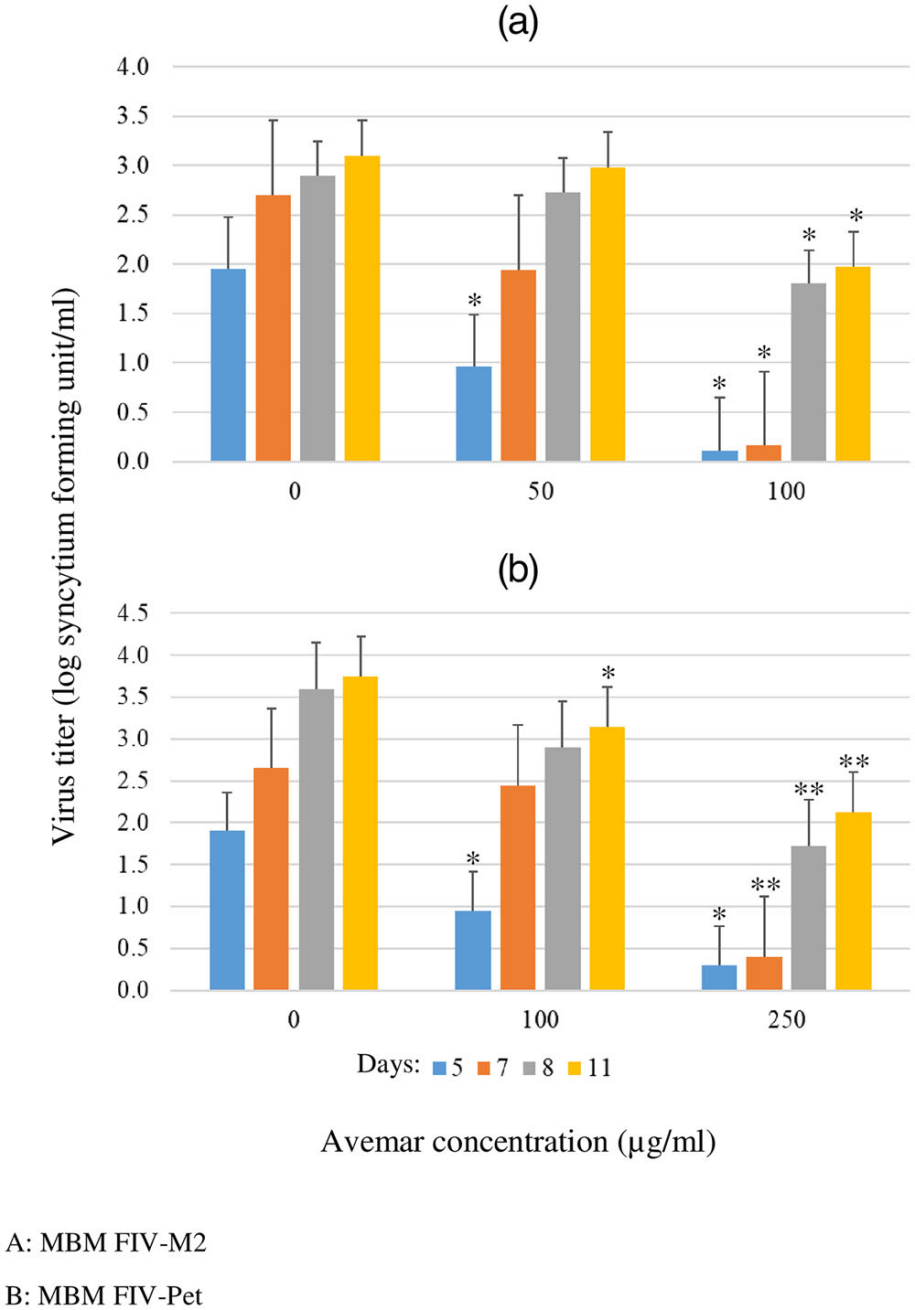
Quantitation of the residual feline immunodeficiency virus (FIV) infectivity in Avemar‐treated MBM cells: (A) FIV Pisa‐M2 (FIV‐M2) infection; (B) feline immunodeficiency virus‐Petaluma (FIV‐Pet) infection. Supernatant fluids were collected on days 5, 7, 8 and 11 and titrated on Crandell Rees feline kidney (CRFK) cells. *p < 0.05, **p < 0.001.

Growing MBM cells formed small clumps in suspension. By 24‐h pi, using FIV‐M2 or FIV‐Pet, cell clumps became smaller and contained fewer cells, and the individual cells gradually became smaller. By day 10, the majority of cells became very small and dense. Cells completely lysed by day 17. Avemar treatment delayed FIV‐induced cell death in a dose‐dependent fashion. In the early days pi, in FIV‐M2 infected cultures, 10–250 μg/mL AP partially, and 500–1000 μg/mL AP completely inhibited CPE. In FIV‐Pet infected cultures, the same effects were found at 10–750 μg/mL and 1000 μg/mL AP, respectively. Virus production in the supernatants showed a 3–5 log decrease in virus titre depending on AP concentration and time pi. No difference was found between cells infected at −2, −1, 0, 1 or 2 h pi. Results showing residual virus titres on days 5, 7, 8 and 11 are summarized in Figure 2. On subsequent days, CPE induced by FIV‐M2 progressed, but the effect of FIV‐Pet remained static. Minimal difference was observed in the replication of the two viruses (Figure 2A,B). On day 17 pi, CPE was still incomplete in FIV‐Pet‐infected cultures treated with 10–500 μg/mL AP, and CPE was 100% in similarly treated FIV‐M2‐infected cells (data not shown).

### 3.3 Effect of Avemar pulvis on FIV‐Pet‐producing FL‐4 cells

**FIGURE 3:**
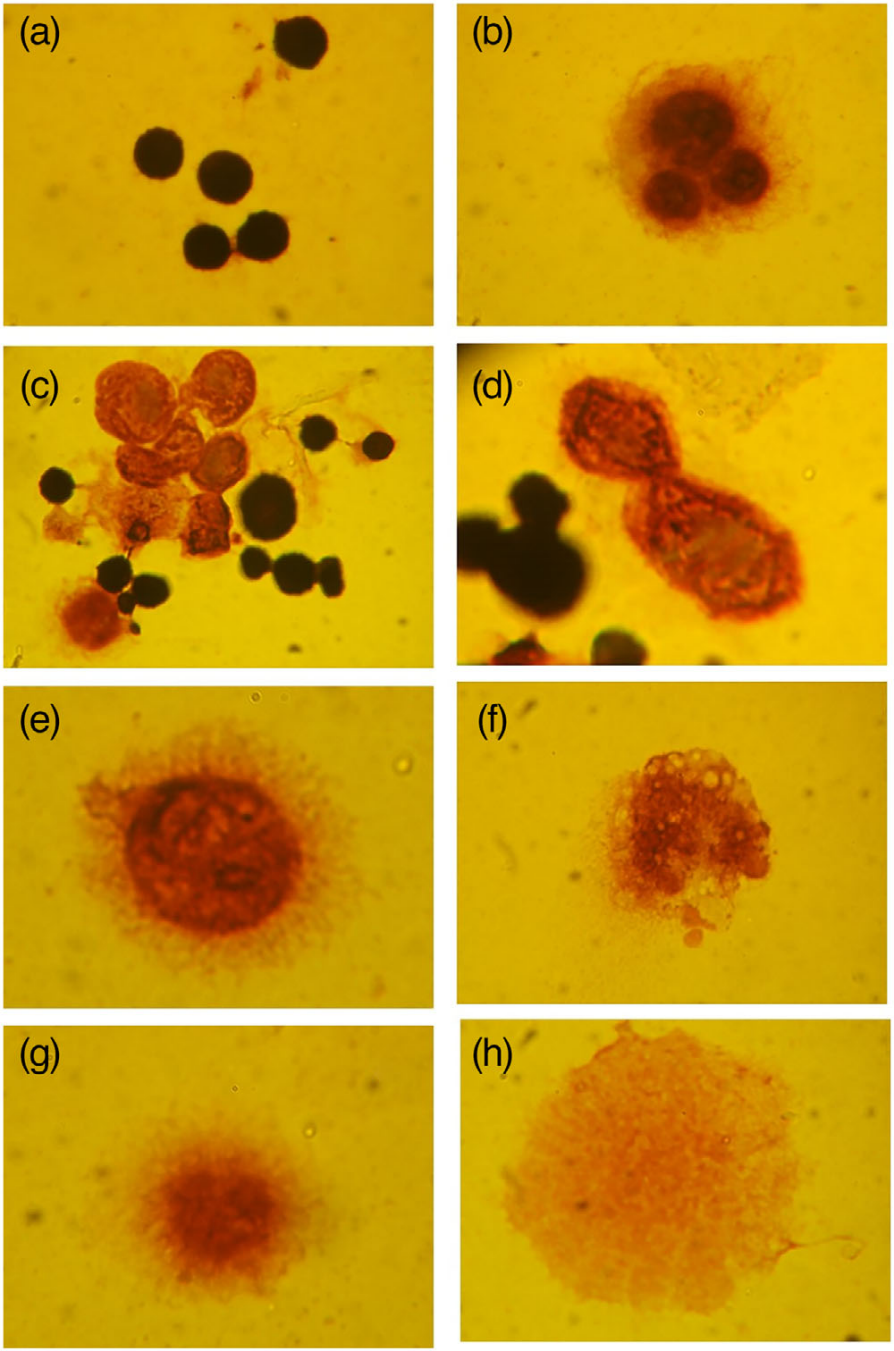
Effect of Avemar on the morphology of feline immunodeficiency virus‐Petaluma (FIV‐Pet) producing FL‐4 cells. Photographs were taken on days 0 (A), 7 (B), 8 (C and D), 9 (E), 10 (F), 11 (G) and 12 (H). Original magnification was 200×.

**FIGURE 4:**
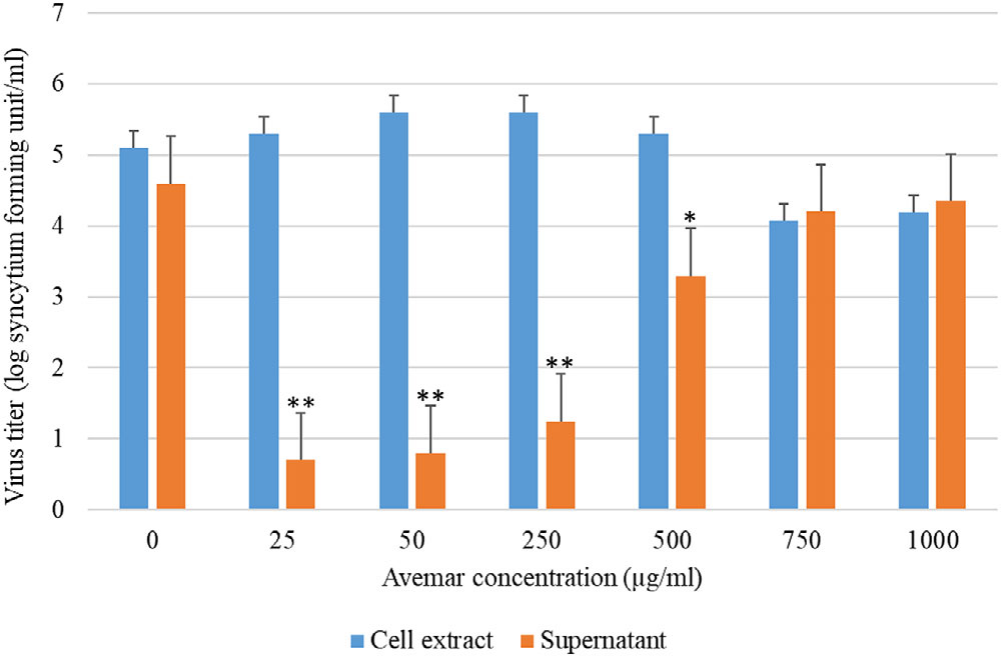
Quantitation of the residual feline immunodeficiency virus‐Petaluma (FIV‐Pet) infectivity in Avemar‐treated FL‐4 cultures. Samples from supernatant fluids and disrupted cells were taken on day 4 after Avemar treatment. Residual infectivity was titrated on Crandell Rees feline kidney (CRFK) cells. *p < 0.05, **p < 0.001.

Avemar treatment affected immortalized FL‐4 cells in a dose‐dependent and time‐dependent fashion. FL‐4 cells exhibit regular lymphocyte morphology (Figure 3A). For Avemar concentrations of 25–1000 μg/mL, intercellular cytoplasmic bridges were increasingly observed until day 8 (Figure 3C,D), subsequently followed by multinucleate giant cell formation (Figure 3B). Cell membrane blebbing, cytoplasmic granularity, chromatic clumping and cell shrinkage were observed, followed by the complete disintegration of the nuclei (Figure 3E–G). Finally, the cytoskeleton of cells floated in the culture medium as loose clouds (Figure 3H). Supernatants of cells treated with low concentrations of AP (25–250 μg/mL) contained very small amounts of FIV, whereas supernatants of cells treated with high concentrations (500–1000 μg/mL) contained large amounts of virus. All cell lysates treated with AP concentrations between 25 and 250 μg/mL contained a high amount of infectious FIV‐Pet (Figure 4).

### 3.4 Effect of Avemar pulvis on FIV‐infected CRFK cells

**FIGURE 5:**
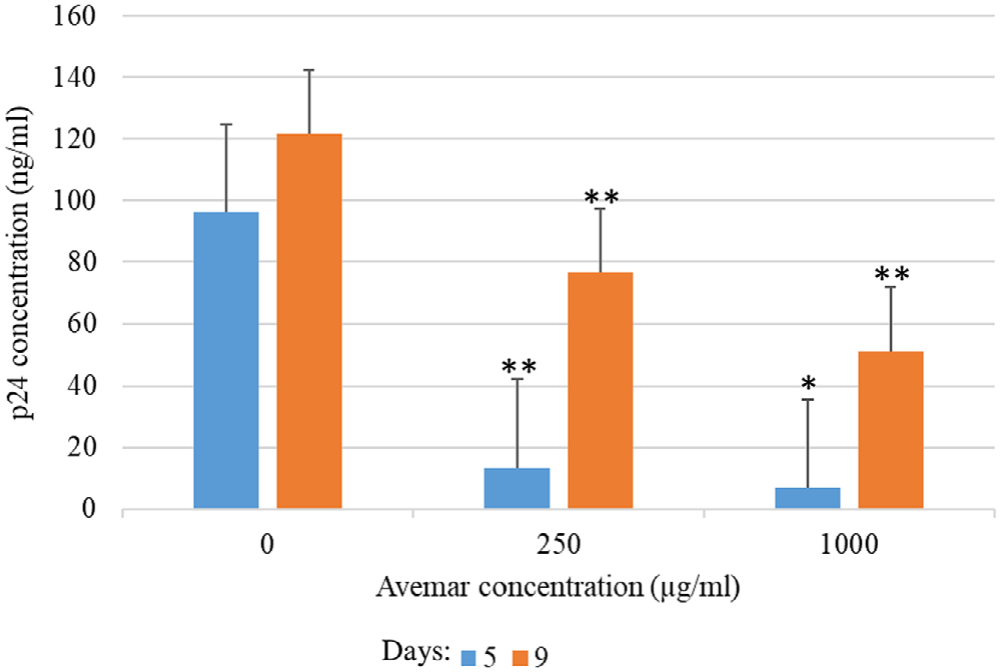
The effect of Avemar on feline immunodeficiency virus‐Petaluma (FIV‐Pet) p24 antigen production by Crandell Rees feline kidney (CRFK) cells. Antigen concentration in the supernatants fluids was determined according to the standards of the kit. *p < 0.05, **p < 0.001.

AP concentrations of 250–1000 μg/mL transiently delayed the onset and progression of CPE in a dose‐ and time‐dependent manner (data not shown). Virus production was quantified in supernatants harvested at 0, 5 and 9 days pi by measurement of p24 content. The effect of representative low and high AP treatments is shown in Figure 5.

### 3.5 Effect of Avemar pulvis on FeAdV replication in HeLa and CRFK cells

**FIGURE 6:**
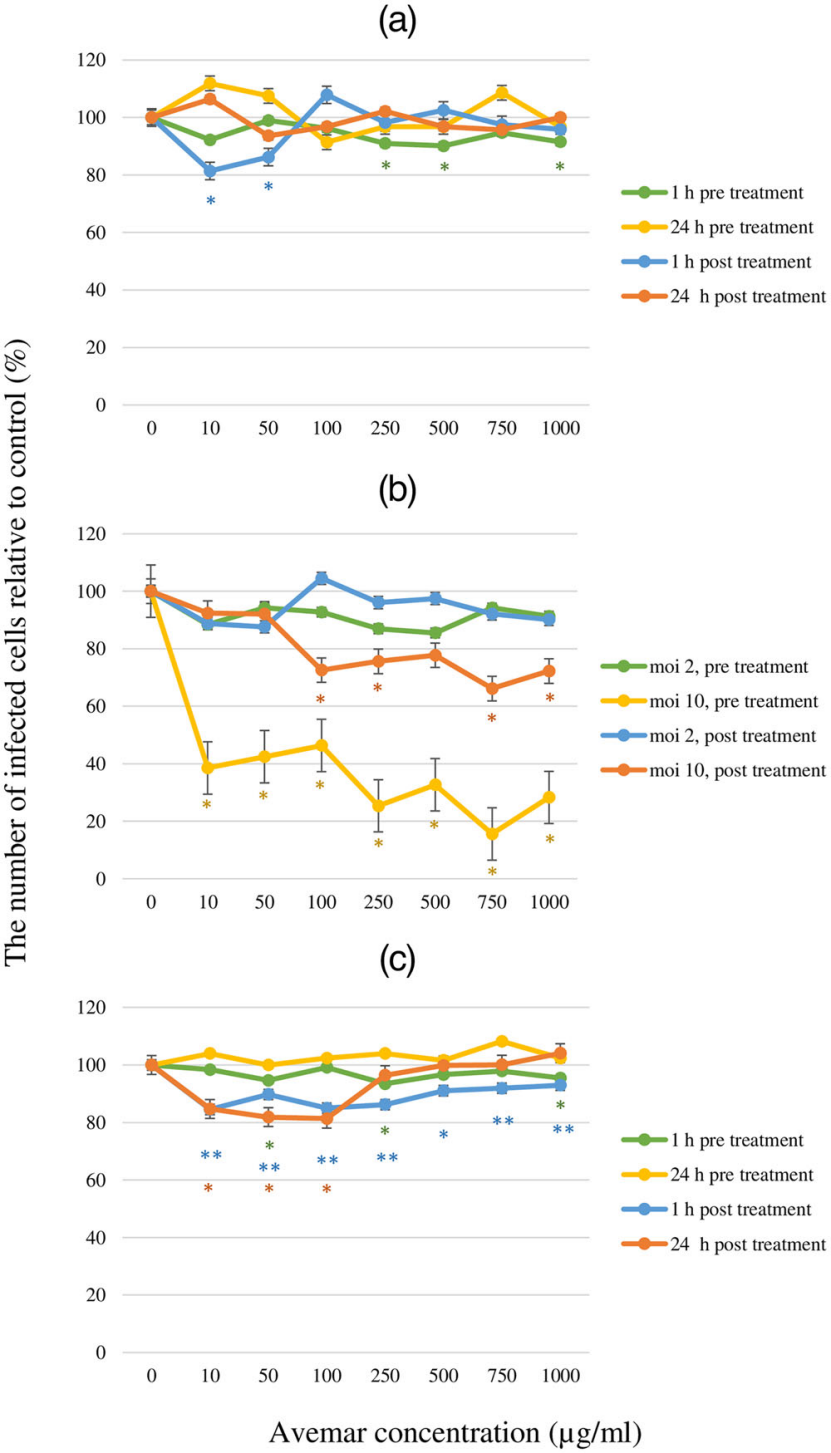
Effect of Avemar on the intracellular feline adenovirus (FeAdV) hexon antigen production in Crandell Rees feline kidney (CRFK) and HeLa cells: (A) Avemar pre‐ and post‐treatment of CRFK cells at 1 and 24 h. Multiplicity of infection (moi) was 1; (B) Avemar pre‐ and post‐treatment of CRFK cells at 24 h. FeAdV input was moi 2 and 10; (C) Avemar pre‐ and post‐treatment of HeLa cells at 1 and 24 h. FeAdV input was moi 1. The ratio of hexon antigen positive cells was determined on day 3 post‐infection. *p < 0.05, **p < 0.001.

**FIGURE 7:**
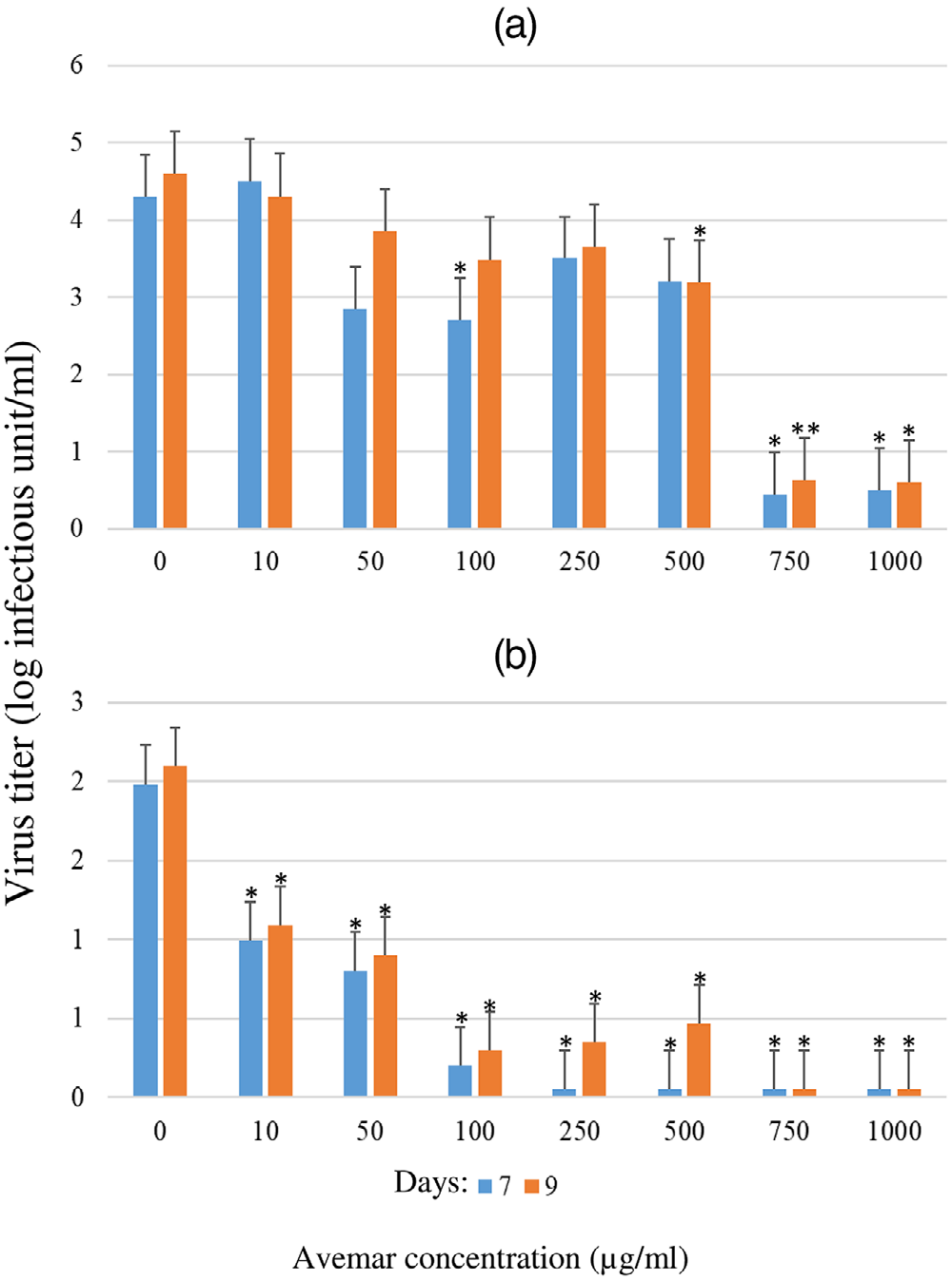
Quantitation of the residual feline adenovirus (FeAdV) infectivity in the supernatant fluids of Avemar‐treated Crandell Rees feline kidney (CRFK) cells. Avemar treatment preceded infection by 24 h. FeAdV inputs were: (A) moi 1; (B) moi 0.1. Samples were collected on day 3 post‐infection, and virus titres were quantitated on days 7 and 9 of cultures. *p < 0.05, **p < 0.001.

Following FeAdV infection, HeLa and CRFK cells untreated with Avemar detached from the tissue culture flasks within 3–5 days; the rounded cells forming small clumps prior to lysis. Following AP treatment, the progression of CPE was delayed in a dose‐ and time‐dependent manor. In low (1 and 2 moi) input FeAdV virus‐infected CRFK cells, Avemar concentrations applied 1 or 24 h before or after infections did not significantly influence FeAdV antigen production (Figure 6A,B). In contrast, 24 h AP pretreatment very strongly inhibited hexon antigen production of high input virus (10 moi) infected cultures in a dose‐dependent manner. Post‐infection AP treatment also exerted a significant inhibitory effect, though, to a lesser extent (Figure 6B). FeAdV replication following low input (moi 1 and 0.1) was very strongly inhibited by increasing AP concentrations when cells had been 24 h pretreated with the drug (Figure 7A,B). FeAdV hexon antigen production by HeLa cells was hardly influenced by AP pre‐ or post‐treatment using low or high input virus (Figure 6C).

## 4 DISCUSSION

This study is the first in‐depth report on the effect of Avemar on viruses. We studied the effect of Avemar on feline lymphoid and epithelial cells. Cytotoxicity of a single dose of AP on feline cells was apparent between 1000 and 2000 μg/mL AP concentration. Low concentrations of AP stimulated the growth of MBM cells while cell growth was strongly inhibited at higher concentrations of AP. The growth of FL‐4‐Pet cells exhibited a similar pattern, although the stimulatory effect of low AP concentrations was delayed beyond day 3. The mechanism of the delay is still to be explored in future experiments. Following AP treatment, the viability and replication of CRFK cells remained diminished transiently. A small fraction of these cells survived and regained replicative capacity, perhaps parallel to the degradation of AP. In contrast, in the early days of treatment, HeLa cultures seemed to be resistant to all AP concentrations. However, the delayed effect of AP irreversibly damaged their viability and replication. Our observations correspond with the apoptotic effect of Avemar on malignant human cells (Zhurakivska et al., 2018).

Infection of MBM cells was used to model acute FIV‐M2 and FIV‐Pet infection, the former having been previously shown to replicate at higher titres (Matteucci, et al., 1995, Tarcsai et al., 2021). A single dose of Avemar strongly inhibited the CPE effect of acute FIV infection with a simultaneous reduction in the viral load of the media. FIV‐M2 appeared to be slightly more sensitive to the antiviral activity of AP than FIV‐Pet. Earlier studies have shown that FIV‐M2‐infected MBM cells lysed without syncytia formation (Matteucci et al., 1995), and we have previously shown that acute FIV‐M2 infection of MBM cells elicits programmed cell death (Tarcsai et al., 2021). FIV is known to induce apoptosis of acutely infected, IL‐2‐dependent T‐lymphoid MYA‐1 cells (Ohno et al., 1994) and peripheral blood lymphocytes of cats (Bishop et al., 1993).

FL‐4 cells continuously producing FIV‐Pet were used to model chronic FIV infection. The effect of Avemar in FL‐4‐Pet cells showed a biphasic pattern depending on the concentration of the compound. Lower concentrations of Avemar very strongly prevented the release of FIV into the environment, whereas higher concentrations disintegrated infected cells, causing the deliberation of membrane‐bound particles and their flow into the medium. In the case of both MBM and FL‐4 cells, Avemar might interfere with the budding process. It has been shown that during the late stages of HIV‐1 replication, immature virus particles are assembled at the plasma membrane interface and released by budding. Recently, myo‐inositol hexakisphosphate (IP6 or phytic acid, phytate) has been identified as an essential cofactor of this budding process, namely the IP6‐driven HIV‐1 viral protein (Gag) assembly has been shown a mandatory mechanistic part of HIV‐1 virion budding (Pak et al., 2022). IP6 is widespread in cereal grains (Hidvégi & Lásztity, 2002). Wheat germ, which is the raw material of Avemar manufacturing, may contain up to 3.9% of IP6 (Schlemmer et al., 2009). Fermentation has been shown to degrade the hexa form of phytic acid by increasing phytase activity, resulting in the generation of lower myo‐inositol polyphosphates (IP5, IP4, IP3, IP2, IP1) and also myo‐inositol (Kumar & Anand, 2021). Consequently, Avemar, the fermentation product of wheat germ, contains no IP6, only its fragments (unpublished data). Although the exact mechanism is still to be explored, we assume that phytate fragments in AP may be factors in the anti‐budding mechanism of Avemar via allosteric inhibition of Gag assembly. Thus, the cofactor role of IP6 in HIV morphogenesis may be impeded by AP. Further support of this proposed mechanism regarding the inhibition of HIV budding came from a study in which a synthetic myo‐inositol pentakisphosphate derivative (L‐HIPPO) was shown to intercept Gag protein assembly, thus inhibiting HIV‐1 virion budding. This mechanism of HIV eradication in reservoir cells has been named ‘lock‐in and apoptosis’, because the ‘locked‐in’ HIV induces apoptosis of the host cells (Tateishi et al., 2017).

Avemar treatment induces cellular death in both acute and chronically infected cells, and the associated changes in cell morphology suggest that this could be due to apoptosis. A previous attempt to induce apoptosis of FL‐4 cells was unsuccessful (Kakizaki et al., 2006). Alpha‐amanitin inhibited cell proliferation and FIV‐Pet production by FL‐4 cells (Tanabe et al., 2021) and also inhibited adenovirus replication (Ledinko, 1971), but, due to its toxicity in vivo, clinical application is impossible.

In CRFK cells, Avemar simultaneously inhibited p24 antigen production and FIV virus release in a dose‐dependent manner. Avemar treatment 24 h pre‐ and post‐infection also interfered with FeAdV replication. Comparison of FeAdV antigen production and virion release indicates that Avemar does not inhibit the gradual accumulation of hexon antigens but inhibits the release of virus particles from the infected cells. Replication of FIV and its release by budding is considerably different from the replication of AdVs which are released from lysed cells. Our results may also have possible clinical relevance because the in vivo production of FIV and FeAdV as well as their carrier cells are inhibited by a sole therapeutic agent (AP). Adenovirus E3 gp19 protein inhibits the expression of MHC class I molecules. Avemar and one of its compounds, 2,6‐DMBQ, also downregulate MHC class I expression in tumour T and B cell lines, selectively promoting their apoptosis (Fajka‐Boja et al., 2002). In HeLa cells, hexon antigen production and virus release were not restricted by AP. HeLa cells harbour integrated human papillomavirus (HPV) genes. It has been shown that E6 and E7 gene products of HPV inhibit apoptosis (Tan et al., 2012) and transactivate adenovirus (Howley et al., 1989) thus counteract the suppressive effects of AP on FeAdV production. Independently from adenovirus production, HeLa cells can be destroyed by Avemar (Imir et al., 2018).

Here we have shown that Avemar inhibited virus release from acutely infected and chronic carrier cells, eventually resulting in cell death, similar to the previously proposed ‘lock‐in and apoptosis’ mechanism. As mentioned, a synthetic inositol pentakisphosphate compound strongly inhibited virus budding, and HIV particles remained trapped in the cells, which subsequently underwent apoptosis (Tateishi et al., 2017). Delivery of this non‐natural compound to the cells needs intermediate agents, and apoptosis of surrounding normal cells was observed (Tateishi et al., 2017). On the contrary, Avemar has been used in human clinical practice without side effects for several decades.

Extrapolating the fate of FIV‐Pet‐producing FL‐4 cells to chronic HIV‐infected humans and FIV‐infected cats has clinical relevance. Chronically lentivirus‐producing cells divide, and descendants indefinitely produce virus‐evading apoptosis. One of the most important observations in our study is that Avemar interrupts the replication of such cells. Upon prolonged treatment with Avemar, their number might gradually decrease, and finally, these cells could be eradicated from the body. In accordance with our results, all these data suggest that, at least in vitro, chronic retrovirus infection can also be targeted at multiple molecular ways by Avemar.

There are some limitations of this study. We could apply only a single dose of Avemar in cell cultures due to the limited life span of cells. Animal experiments would be ideal to maintain a constant Avemar level in the body by using repeated dosages. We could study the chronic infection by using FIV‐Pet‐producing cells only. Unfortunately, no similar cell line is available which can chronically produce a European FIV isolate. As CRFK cells are permissive to both FIV and FeAdV infections, it would be logical to infect these cells simultaneously with both viruses to study their possible interaction. And afterwards, the measurement of FIV and FeAdV production could be carried out separately. Earlier, we described FIV‐induced apoptosis in MBM cells. Here we can suppose, based on morphological signs, that Avemar induced apoptosis in FL‐4 cells. In the present study, we did not aim to determine the exact molecular mechanism of Avemar on viruses and virus‐infected cells. This has to be explored by future studies.

## 5 CONCLUSIONS

In summary, we can conclude that Avemar, as a single nutraceutical, possesses beneficial effects in feline AIDS. It inhibited FIV replication and destroyed retrovirus carrier cells (FL‐4). Furthermore, Avemar inhibited FeAdV, a heterologous virus which belongs to the family of Adenoviridae that can transactivate retroviruses and frequently causes lethal opportunistic infections. Extrapolating the fate of FIV‐Pet‐producing FL‐4 cells to that of the HIV‐producing human lymphocytes, FIV‐infected cats may have important clinical relevance to HIV‐infected humans. Chronically lentivirus‐producing cells divide, and descendants also continuously produce virus. This indefinitive process exists in vivo and can be established in vitro, such as in FL‐4 cells. Cells chronically producing retroviruses evade apoptosis. One of the most important observations in our study is that Avemar interrupts the replication of such cells. It may thus cautiously be supposed that prolonged Avemar treatment will gradually reduce and finally eradicate these retrovirus‐producing cells in the host. If we suppose that AP inhibits FIV and FeAdV, we may also suppose that this nutraceutical may also inhibit HIV and human AdVs. Thus, these aspects ought to be explored in future studies. The latter should include clinical trials with HIV‐infected humans receiving HAART and adjunctive Avemar, as well as clinical studies with FIV‐infected pets and endangered large cats.

## AUTHOR CONTRIBUTIONS

*Conceptualization*: József Ongrádi, Máté Hidvégi, Dharam V. Ablashi. *Formal analysis*: Katalin Réka Tarcsai, Anna Anoir Abbas. *Funding acquisition*: József Ongrádi. *Investigation*: Katalin Réka Tarcsai, Anna Anoir Abbas, Valéria Kövesdi. *Methodology*: Károly Nagy, Máté Hidvégi, Dharam V. Ablashi. *Project administration*: Valéria Kövesdi. *Resources*: Valéria Kövesdi. *Supervision*: József Ongrádi, Máté Hidvégi, Dharam V. Ablashi. *Validation*: Károly Nagy. *Visualization*: Katalin Réka Tarcsai, Anna Anoir Abbas. *Writing – original draft preparation*: Katalin Réka Tarcsai, Károly Nagy, Valéria Kövesdi. *Writing – review and editing*: József Ongrádi, Máté Hidvégi, Dharam V. Ablashi. All authors reviewed and approved the final version of the manuscript.

## CONFLICT OF INTEREST STATEMENT

Dr. Máté Hidvégi received a consultation fee from Biropharma Kft. All the other authors do not declare any conflict of interest.

### PEER REVIEW

The peer review history for this article is available at https://publons.com/publon/10.1002/vms3.1141.

## Data Availability

All relevant data are within the paper.
