# Supplementary material for: The effects of Avemar treatment on feline immunodeficiency virus infected cell cultures
Source: Vet Med Sci. 2023 Apr 20;9(4):1446–55. doi: 10.1002/vms3.1141 (PMC10357279; doi:10.1002/vms3.1141)
Supplement: Supplementary file 1 [file VMS3-9-1446-s001.docx]

Images of the organisms

**Figure S1**

vms31141-sup-0002-SuppFigure.jpg

Feline immunodeficiency virus particles in the vesicles of a CRFK cell. Original magnification: x21,000. Made by József Ongrádi MD, PhD and Professor Sándor Paku, DSc (1st Department of Pathology and Experimental Cancer Research, Semmelweis University, Budapest, Hungary)

**Figure S2**

vms31141-sup-0003-SuppFigure.jpg

Feline adenovirus particles in a disintegrating CRFK cell Original magnification: x9800 . Made by József Ongrádi MD, PhD and Professor Sándor Paku, DSc (1st Department of Pathology and Experimental Cancer Research, Semmelweis University, Budapest, Hungary)
