# Supplementary figures and images for: The effects of Avemar treatment on feline immunodeficiency virus infected cell cultures
Source: Vet Med Sci. 2023 Apr 20;9(4):1446–55. doi: 10.1002/vms3.1141 (PMC10357279; doi:10.1002/vms3.1141)

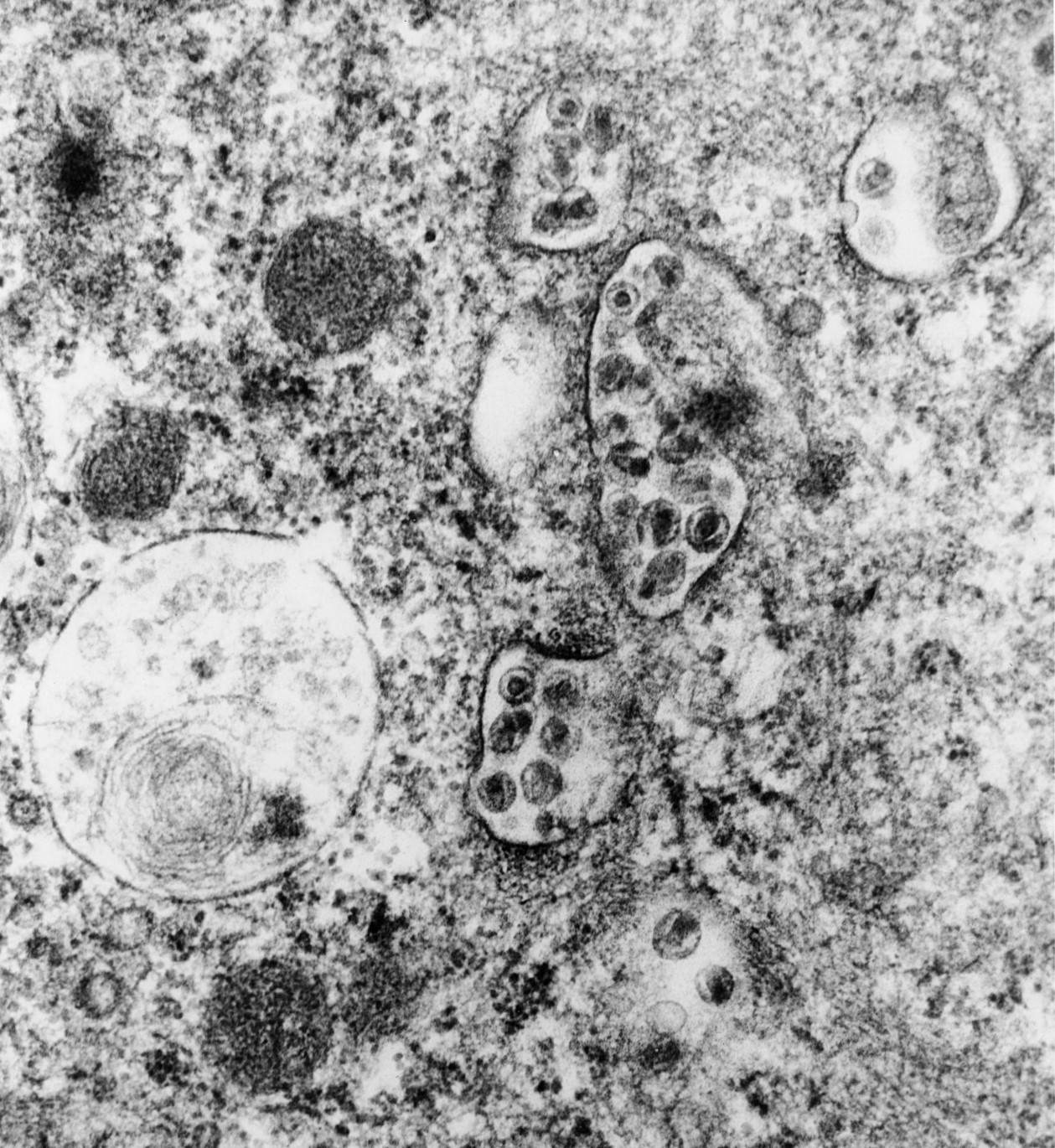

Supplement: Supplementary file 2 [file VMS3-9-1446-s003.jpg]

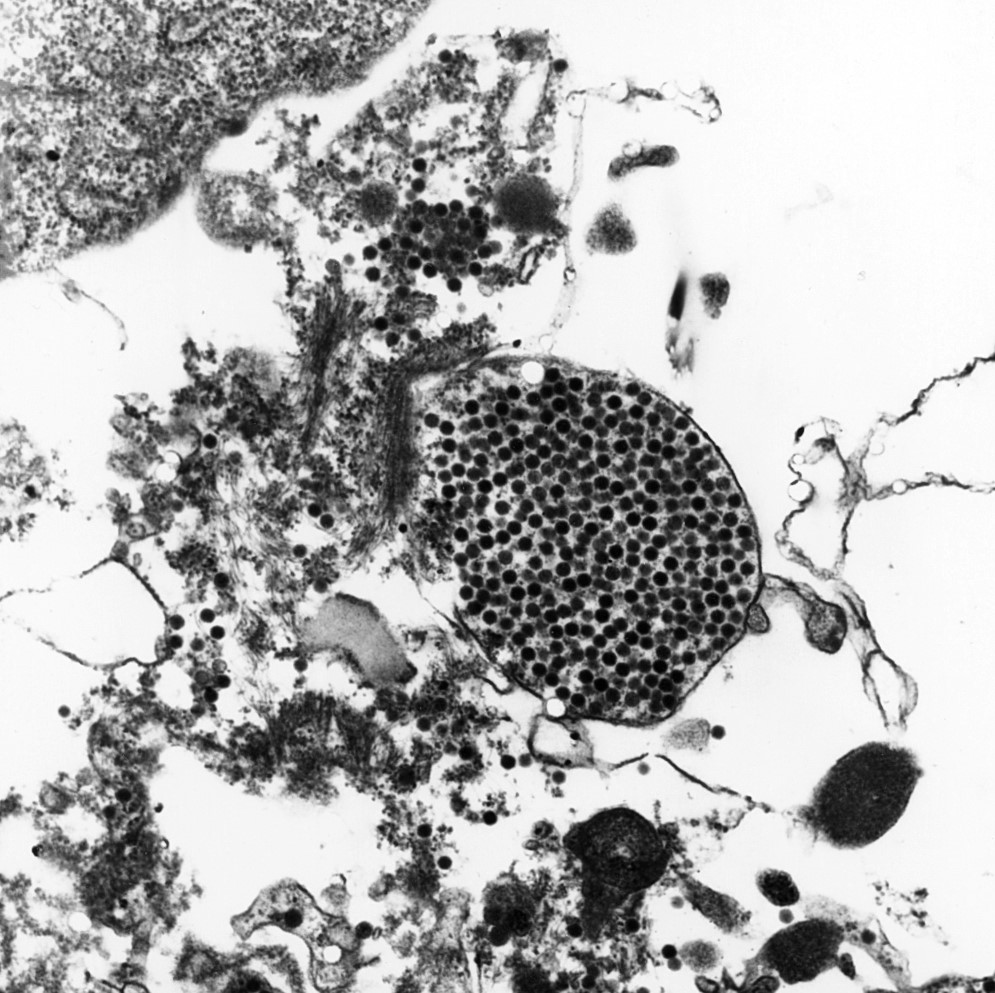

Supplement: Supplementary file 3 [file VMS3-9-1446-s002.jpg]
